# MazEF Homologs in *Symbiobacterium thermophilum* Exhibit Cross-Neutralization with Non-Cognate MazEFs

**DOI:** 10.3390/toxins16020081

**Published:** 2024-02-03

**Authors:** Yu-Nong Jiang, Hiroko Tamiya-Ishitsuka, Rie Aoi, Takuma Okabe, Akiko Yokota, Naohiro Noda

**Affiliations:** 1 Master’s/Doctoral Program in Life Science Innovation, Graduate School of Comprehensive Human Sciences, University of Tsukuba, Tsukuba 305-8572, Ibaraki, Japan; 2 Biomedical Research Institute, National Institute of Advanced Industrial Science and Technology (AIST), Tsukuba 305-8566, Ibaraki, Japan; 3 Department of Life Science and Medical Bioscience, Waseda University, Shinjuku-ku 162-8480, Tokyo, Japan; 4 Master’s/Doctoral Program in Life Science Innovation, School of Integrative and Global Majors, University of Tsukuba, Tsukuba 305-8572, Ibaraki, Japan

**Keywords:** *Symbiobacterium*, toxin–antitoxin systems, MazF, RNase, horizontal gene transfer

## Abstract

Toxin–antitoxin systems are preserved by nearly every prokaryote. The type II toxin MazF acts as a sequence-specific endoribonuclease, cleaving ribonucleotides at specific sequences that vary from three to seven bases, as has been reported in different host organisms to date. The present study characterized the MazEF module (MazEF-sth) conserved in the *Symbiobacterium thermophilum* IAM14863 strain, a Gram-negative syntrophic bacterium that can be supported by co-culture with multiple bacteria, including *Bacillus subtilis*. Based on a method combining massive parallel sequencing and the fluorometric assay, MazF-sth was determined to cleave ribonucleotides at the UACAUA motif, which is markedly similar to the motifs recognized by MazF from *B. subtilis* (MazF-bs), and by several MazFs from Gram-positive bacteria. MazF-sth, with mutations at conserved amino acid residues Arg29 and Thr52, lost most ribonuclease activity, indicating that these residues that are crucial for MazF-bs also play significant roles in MazF-sth catalysis. Further, cross-neutralization between MazF-sth and the non-cognate MazE-bs was discovered, and herein, the neutralization mechanism is discussed based on a protein-structure simulation via AlphaFold2 and multiple sequence alignment. The conflict between the high homology shared by these MazF amino acid sequences and the few genetic correlations among their host organisms may provide evidence of horizontal gene transfer.

## 1. Introduction

Toxin–antitoxin (TA) systems are highly conserved among prokaryotes and prevalent in nearly every prokaryotic chromosome [1]. These systems are composed of a toxin protein and a cognate antitoxin, with the latter counteracting the toxicity of the former. According to the neutralization mechanism, TA systems are divided into eight types [2]; among them, type II TA systems are characterized by a pair of proteins where the labile antitoxin binds to the toxin protein for neutralization.

The most studied TA system, MazEF, is a typical type II module. The *mazEF* locus consists of one operon, and *mazE* is commonly located upstream of the *mazF* locus. In normal-growing cells, one MazE dimer captures two MazF dimers in the form of (MazF)_2_-(MazE)_2_-(MazF)_2_ heterohexamer [3]. MazE not only binds to MazF, blocking the active site, but also weakly binds upstream of the *mazEF* locus to repress its own transcription. While MazF enhances this autoregulation through cooperative binding, it also manipulates its own expression by cleaving the transcript of itself during recovery from the stress caused by MazF activation [4,5].

Identified first in *Escherichia coli*, the MazF toxin (MazF-ec) is released when the proteolytically labile MazE (MazE-ec) is readily degraded by the ATP-dependent proteases ClpP and Lon [6,7]. The liberation of MazF-ec is triggered under various stress conditions, including antibiotics, thymine-less death, high temperatures, DNA damage, and oxidative stress [8,9,10]. The unrestricted MazF-ec cleaves at the ACA motif on ssRNA and generally digests cellular transcripts and/or rRNA precursors [11,12].

Nonetheless, this global digestion of transcripts has not been characterized in other MazF host cells due to differences in recognition sequences. To date, more than ten different RNA sequences from three to seven bases have been reported to be specifically cleaved by various MazFs. The unique sequences have been particularly identified from Gram-negative bacteria and archaea, such as UGG cleaved by MazF conserved in *Nitrosomonas europaea* [13], and a unique seven-base motif UUACUCA cleaved by MazF from *Haloquadra walsbyi* [14]. The specific recognition has also been proposed to be decided by several amino acid residues. Arg25 and Thr48 of MazF from *Bacillus subtilis* (MazF-bs) are essential, especially for enzymatic activity [15]; moreover, the mutation into Asn36 of MazF from *Candidatus* Desulforudis audaxviator shifted the recognition sequence [16]. Yet notably, as represented by MazF-bs, several MazFs from Gram-positive bacteria have been discovered successively to recognize and cleave at the exact same UACAU motif [17,18,19,20]. An analysis conducted through BLASTCLUST unveiled the distribution of 102 homologs of MazF-bs across 99 strains, with 97 belonging to Gram-positive bacteria. The homology between these MazFs stands in contrast to the unpredictable scattering of their host bacteria on the 16S rRNA phylogenetic tree [17]. Furthermore, the neutralization of MazF-bs by non-cognate MazE-ec, which shared low homology to MazE-bs, was also examined by a growth assay. The resulting growth inhibition caused by MazF-bs in *E. coli* cells was neutralized by MazE-bs, but not eased by MazE-ec [17].

*Symbiobacterium thermophilum*, a Gram-negative syntrophic bacterium, was first obtained at 60 °C with a thermophilic *Bacillus* strain S, which was identified two decades later as *Geobacillus stearothermophilus* through phylogenetic analysis [21,22]. Despite having a syntrophic property proposed to be caused by the genetic defect of carbonic anhydrase [23], the bacterium showed a wide occurrence in the environment throughout soils and fertilizers. The efficient proliferation of *S. thermophilum* in mixed culture with *B. subtilis*, *Thermus thermophilus,* or *G. stearothermophilus* indicated multiple supporting species in natural surroundings. Genomic studies of *S. thermophilum* have suggested the inclusion of mobile elements in its genome [24]. On the chromosome of the *S. thermophilum* IAM 14863 strain, the toxin–antitoxin database (TADB 2.0) predicted two type II TA loci, one of which was recognized as a *mazEF* locus homolog (*mazEF-sth*) (Dataset S1, S2) [25].

The present study involved stipulating the specific cleavage of *S. thermophilum* MazF (MazF-sth) and assessing its ribonuclease activity via a method comprising a combination of massive parallel sequencing and a fluorometric assay based on the fluorescence resonance energy transfer (FRET) phenomenon. Subsequently, its physiological role was predicted. The conserved amino acid residues essential for MazF-bs catalysis were then investigated to elucidate their role in the enzymatic property of MazF-sth. In addition, the cross-neutralization between MazEF-sth and MazEF-bs was examined, focusing on MazF with the non-cognate MazE from different organisms. Herein, the possibility of the *mazEF* locus being a module that can be acquired by horizontal gene transfer is also discussed.

## 2. Results

### 2.1. MazEF-sth Conserved in S. thermophilum Functions as a Ribonuclease and Inhibitor

**Figure 1:**
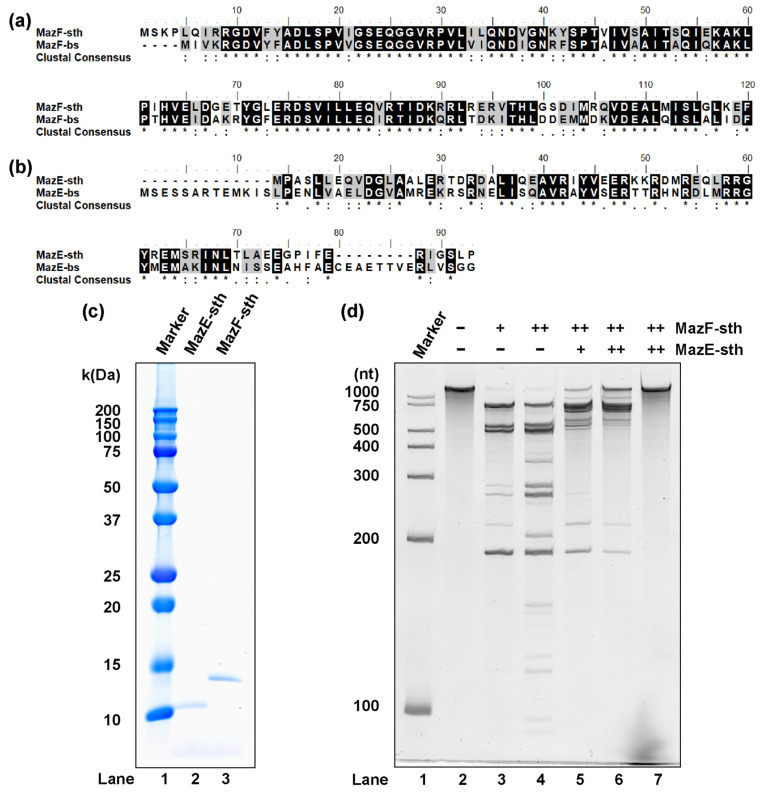
Identification of presumed MazEF-sth. Pairwise alignments between (**a**) MazF-sth with MazF-bs and (**b**) MazE-sth with MazE-bs. Identical amino acids are shadowed in black, and highly similar amino acids are shadowed in gray. An asterisk (*) indicates a position with a fully conserved residue. A colon (:) indicates a highly conserved position between groups of amino acids with strongly similar characteristics. A period (.) indicates a conserved position between groups of weakly similar properties. (**c**) The molecular weight and purity of MazEF-sth were analyzed by SDS-PAGE. Lane 1, marker; lane 2, MazE-sth (theoretical molecular weight: 9.8 kDa); lane 3, MazF-sth (14.9 kDa). (**d**) The ribonuclease activity of MazF-sth and the inhibition of MazE-sth. Synthetic RNA 1500-1 (1533 nt) was incubated at 37 °C for 90 min with MazF-sth and/or MazE-sth. Lane 1, marker; lane 2, negative control with no enzyme; lane 3, 0.5 pmol of MazF-sth; lane 4, 5 pmol of MazF-sth; lane 5, 5 pmol of MazF-sth pre-mixed with 10 pmol of MazE-sth; lane 6, 5 pmol of MazF-sth pre-mixed with 50 pmol of MazE-sth; and lane 7, 50 pmol of MazE-sth.

Via an operon-based structure search carried out by using TADB 2.0, the gene locus of STH2934-STH2935 was presumed to encode type II TA system MazEF (MazEF-sth), mazE-sth located 14 bases upstream from mazF-sth [25]. As the growth of the origin organism *S. thermophilum* is particularly supported by the host *B. subtilis* of MazEF-bs in mixed culture, pairwise sequence alignment between MazEF-bs and MazEF-sth was performed. The putative MazF-sth protein consists of 120 amino acids, exhibiting 65.0% high identity and 85.8% similarity to MazF-bs (Figure 1a). Comparatively, MazE-sth has 72 amino acids, exhibiting 31.2% identity with MazE-bs (Figure 1b). To discuss whether the two homologs in *S. thermophilum* function as a type II TA module, the presumed proteins were expressed with the *E. coli* expression system and purified based on affinity chromatography (Figure 1c).

The synthetic RNA substrate was treated with MazF-sth at 37 °C; consequently, ribosome-independent ribonuclease activity was observed for MazF-sth (Figure 1d). When MazF-sth was pre-mixed with MazE-sth, a reduction in cleavage activity was seen (Figure 1d); therefore, it was concluded that these two homologs function as the toxin and antitoxin of the type II TA system MazEF. As *S. thermophilum* was first obtained by culture at 60 °C, the performance of MazF-sth was examined across a series of temperatures from 4 °C to 90 °C, revealing that the enzyme showed nearly equal strongest cutting activity from 37 °C to 60 °C (Figure S1).

### 2.2. MazF-sth Preferentially Cleaves the UACAUA Motif

**Figure 2:**
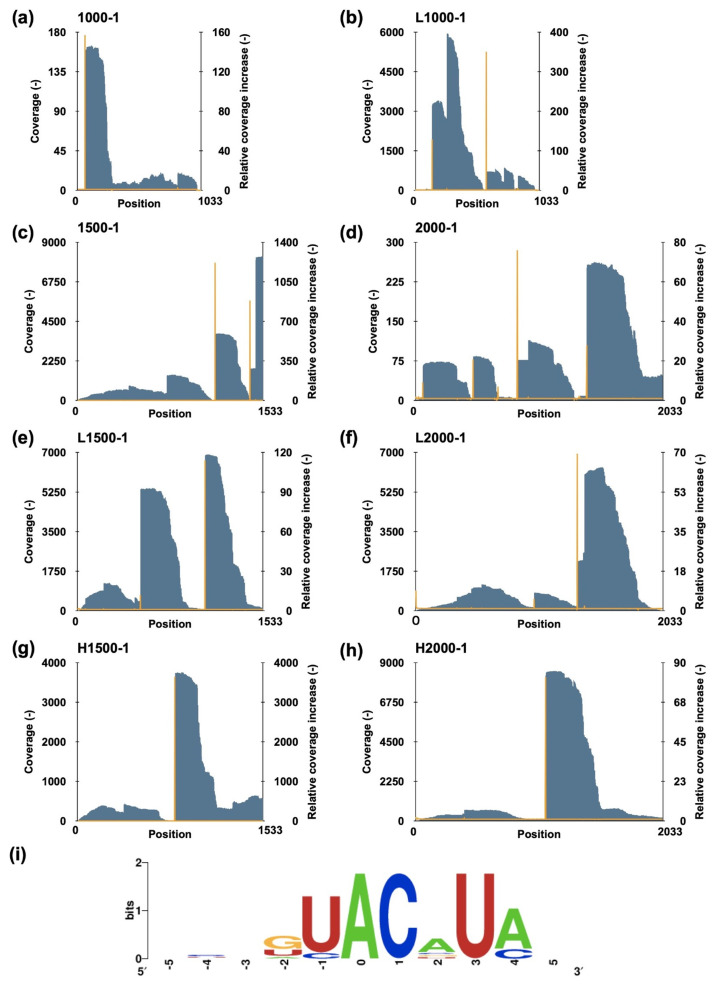
Assessment of the MazF-sth cleavage sequence. Eight synthetic RNAs—(**a**) 1000-1, (**b**) L1000-1, (**c**) 1500-1, (**d**) 2000-1, (**e**) L1500-1, (**f**) L2000-1, (**g**) H1500-1, and (**h**) H2000-1—were digested by MazF-sth. After sequencing, the substrate sequence position was represented as the X-axis in the 5′ to 3′ direction. The coverages of each position (gray bar) are indicated by the left Y-axis, while the RCIs (orange line) are indicated by the right Y-axis. (**i**) Peripheral sequences around the 10 positions with the highest RCIs were aligned, and the conserved sequence was visualized using WebLogo. The site with the highest RCI was set to zero on the X-axis; the presumed cleavage site was between the site of −1 and 0.

A method combining massive parallel sequencing and a fluorometric assay was employed to uncover the cleavage pattern of MazF-sth [26]. A set of eight artificial synthetic RNAs (1000-1, L1000-1, 1500-1, L1500-1, H1500-1, 2000-1, L2000-1, and H2000-1) was fragmented by MazF-sth at 60 °C [27]. The 5′ end of the digested sites was phosphorylated and then ligated with a unique barcode to conserve the digestion sites. Then, the fragments were reverse-transcribed to synthesize the cDNA library and loaded on a MiSeq platform. The sequencing reads were processed using CLC Genomics Workbench version 12.0; the barcoded reads were collected. The barcode sequences were trimmed from these collected reads, and finally, the remaining sequences were mapped according to the known substrate sequences. The coverage at each position was counted and visualized in 5′ to 3′ direction (Figure 2a–h). To estimate the cleavage sites, a parameter relative coverage increase (RCI) was defined as the coverage of the (n)^th^ position divided by the coverage of the (n−1)^th^ position. The 10 sites with the highest RCI, as well as the 11 base sequences centered on them, were analyzed (Table S1). The peripheral sequences of the sites were aligned and visualized using WebLogo version 2.8.2 (Figure 2i) [28]. Ultimately, a consensus sequence U^ACAUA was presumed to be the candidate recognition sequence for MazF-sth (^ indicates cleavage sites). Considering the low consistency observed on the positions of U_1_, A_4_, and A_6_, a fluorometric assay was performed to determine the specific cleavage sequence with a set of fluorescently labeled chimeric oligonucleotide probes (Table S2).

**Figure 3:**
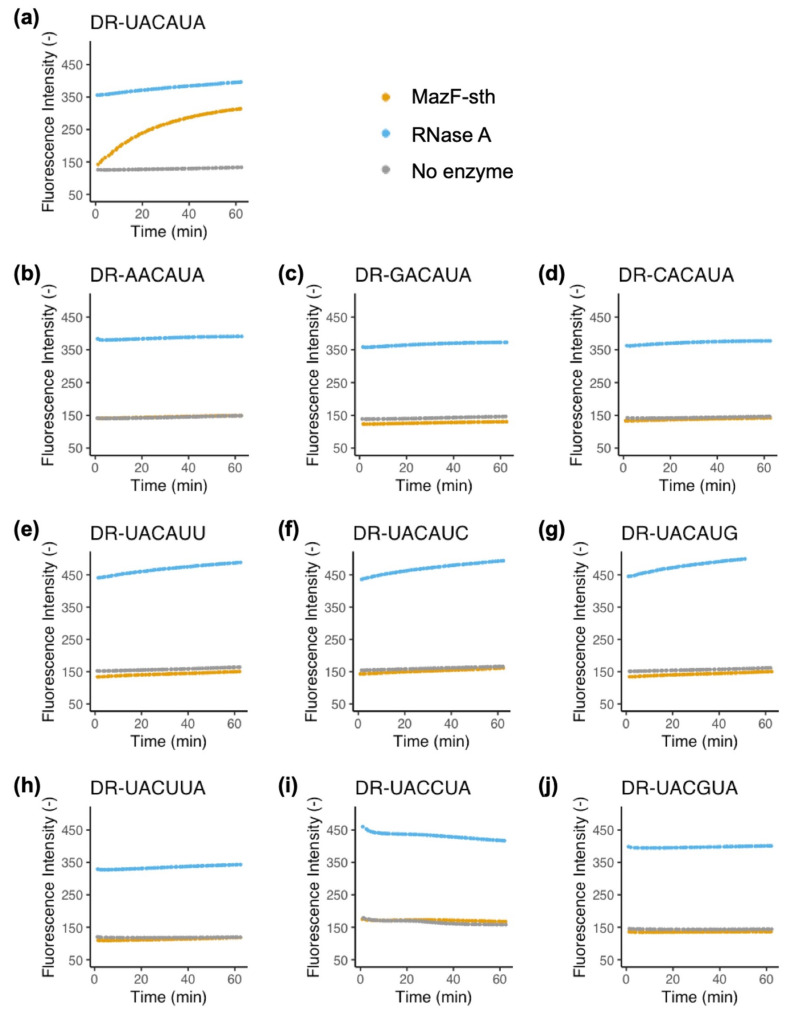
Identification of MazF-sth specific cleavage sequences with fluorescently labeled probes: (**a**) DR-UACAUA, (**b**) DR-AACAUA, (**c**) DR-GACAUA, (**d**) DR-CACAUA, (**e**) DR-UACAUU, (**f**) DR-UACAUC, (**g**) DR-UACAUG, (**h**) DR-UACUUA, (**i**) DR-UACCUA, and (**j**) DR-UACGUA (Table S2). MazF-sth (orange) was incubated with the fluorometric probes at 60 °C for 60 min. Fluorescence intensities in the presence of RNase A (blue) and absence of enzymes (gray) were recorded as the control. Fluorescence intensities were continuously assessed and recorded according to their reactions with each probe.

Decorated with a 6-carboxy fluorescein (6-FAM) at the 5′ end and a black hole quencher (BHQ-1) at the 3′ end, the chimeric oligo probes were synthesized by inserting the estimated cleavage RNA sequences into the center of 10 bases of deoxyadenosine oligos. For example, to determine the specific recognition towards the first position of U_1_, the probes UACAUA, AACAUA, CACAUA, and GACAUA were applied. The less conserved positions A_4_ and A_6_ were investigated in the same way. In total, 10 probes were designed to verify the specific cleavage for MazF-sth (Table S2). Fluorescence intensity was observed in real time (Figure 3). Once the ribonucleotide was cleaved, resulting in the separation of the BHQ-1 from the 6-FAM, the fluorophore was restored. When the concentration of MazF-sth was limited to 0.01 pmol, a notable increase in fluorescence intensity was observed only with the UACAUA probe, whereas the reactants of the other probes showed no observable change in fluorescence intensity during the one-hour incubation (Figure 3). Notably, with 1 pmol MazF-sth, in addition to the undisputed rise in fluorescence with the UACAUA probe, MazF-sth also showed relatively low but equally impressive catalytic activity for UACUUA and UACAUC, as well as slight activity with AACAUA, UACAUG, and UACAUU (Figure S2). The actual working concentration of free MazF-sth in vivo was of great significance when discussing the cleavage patterns among the transcripts. In conclusion, MazF-sth still showed a preference against the UACAUA motif, revealing the high similarity in the target sequences between MazF-sth and MazF-bs, the latter of which has been reported to cleave a UACAU motif [17].

### 2.3. Estimated Target Coding Sequences of MazF-sth

The coding sequences (CDSs) of *S. thermophilum* strain IAM 14863, totaling 3201, were obtained from the NCBI database as of February 2021. The prevalence of the ‘UACAUA’ motif among the cell transcripts was evaluated using a statistical analysis previously drafted [13]. A total of 31 gene CDSs were found to contain one UACAUA motif for each, thus being possible intracellular targets of MazF-sth (Table 1). Among them, two genes (RS16150 and RS07500) with short CDSs encode products that might be related to the process of biofilm formation. The gene RS16150 encodes the Veg family protein to simulate biofilm formation [29]. Another gene, RS07500, encodes the PilZ domain-containing protein that controls pilus; thus, it is related to cell mobility by being a famous c-di-GMP effector which is an essential component in causing bacteria to change from a motile state to a biofilm [30]. In addition, five annotated genes (RS17880, RS05630, RS02655, RS16455, and RS13520) encode products that are related to the cell wall; four annotated genes (RS08320, RS09880, RS07025, and RS12640) encode membrane proteins that are involved with membrane transports; and six genes (RS16105, RS17470, RS09835, RS16160, RS05755, and RS12140) are related to transcription and translation.

### 2.4. The Conserved Sites of Arg29 and Thr52 in MazF-sth Are Essential for Cleavage Activity

As MazF-sth exhibited high identity with MazF-bs in terms of amino acid sequences (Figure 1a,b), the two MazFs also shared similar cleavage sequences (as discussed in Section 2.2). The two residues of MazF-bs, Arg25 and Thr48, were uncovered to be particularly essential for the catalytic process and are highly conserved among a wide range of MazF homologs [15]. Through pairwise alignment, Arg29 and Thr52, corresponding to the two residues (Arg25 and Thr48, as mentioned in MazF-bs), were also found to be conserved in MazF-sth (Figure 1a). Thus, two mutants, R29A MazF-sth and T52A MazF-sth, were designed by replacing the Arg29 and Thr52 on the toxin with alanine via site-directed mutagenesis. Thereafter, the mutants were produced via a cell-free expression system in addition to the wild type for the cleavage activity assay (Figure S3).

**Figure 4:**
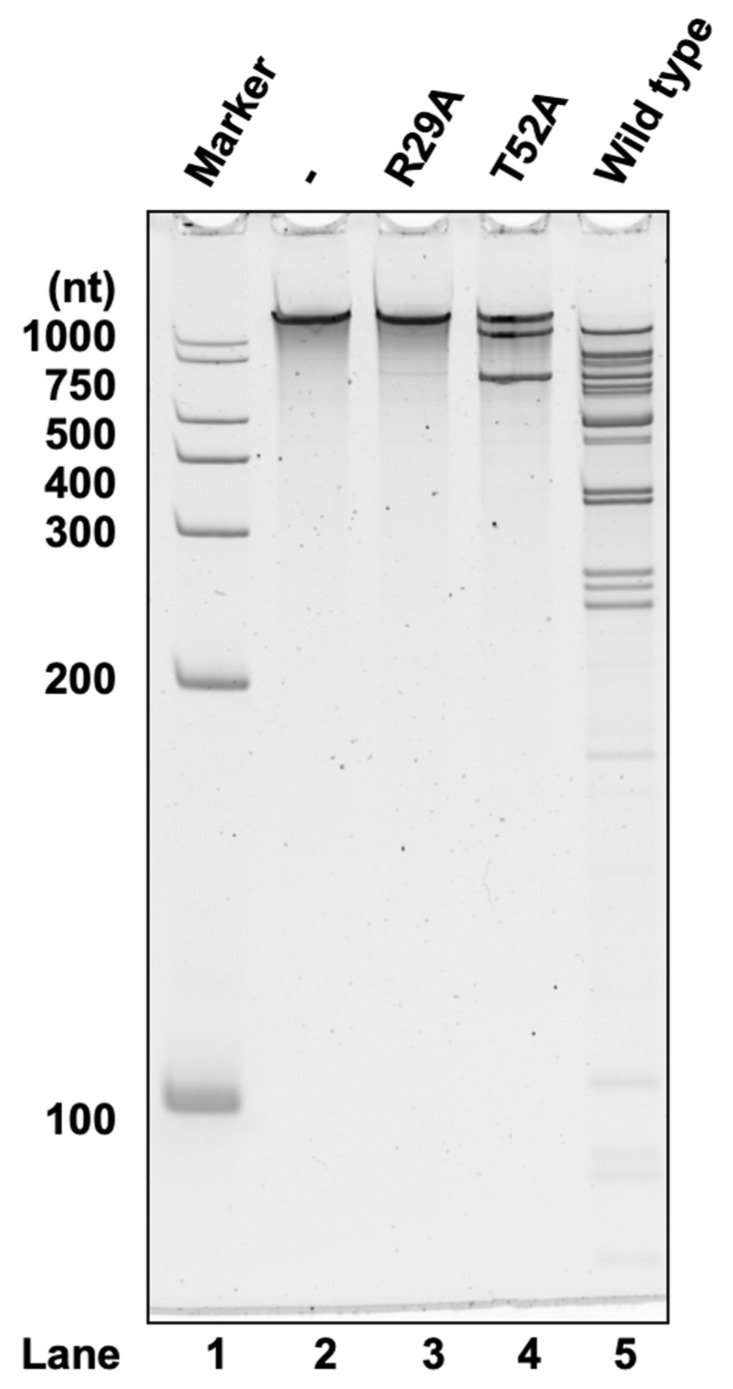
Synthetic RNA substrate 2000-1 (2033 nt) was treated with R29A, T52A, or the wild type MazF-sth. Digested RNA fragments were analyzed by denaturing PAGE. Lane 1, marker; lane 2, negative control with no enzyme; lane 3, R29A MazF-sth; lane 4, T52A MazF-sth; lane 5, wild type MazF-sth.

No fragmentation was seen after the incubation of substrate RNA with R29A (Figure 4). Likewise, the fragment bands were substantially reduced by T52A treatment compared to the wild type (Figure 4). These mutations significantly reduced cleavage activity. Thus, the conserved sites Arg29 and Thr52 are essential for the catalytic activity of MazF-sth.

### 2.5. MazF-sth Enzymatic Activity Was Suppressed by Non-Cognate MazE-bs

**Figure 5:**
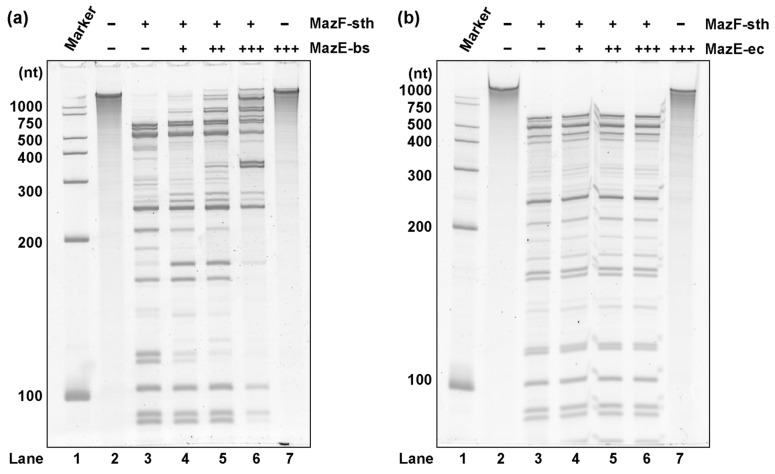
The cross-neutralization of MazF-sth by non-cognate MazEs. The suppression of MazF-sth by non-cognate (**a**) MazE-bs or (**b**) MazE-ec was assessed with synthetic RNA 2000-1 (2033 nt), and the digestion was visualized by denaturing PAGE. One picomole MazF-sth was pre-incubated with 2, 5, or 10 pmol non-cognate MazE. (**a**) The suppression of MazF-sth by non-cognate MazE-bs. Lane 1, marker; lane 2, only substrate RNA without enzyme nor MazE; lane 3, 1 pmol of MazF-sth; lane 4, 1 pmol of MazF-sth and 2 pmol of MazE-bs; lane 5, 1 pmol of MazF-sth and 5 pmol of MazE-bs; lane 6, 1 pmol of MazF-sth and 10 pmol of MazE-bs; lane 7, 10 pmol of MazE-bs. (**b**) The suppression of MazF-sth by non-cognate MazE-ec. Lane 1, marker; lane 2, only substrate RNA without enzyme nor MazE; lane 3, 1 pmol of MazF-sth; lane 4, 1 pmol of MazF-sth and 2 pmol of MazE-ec; lane 5, 1 pmol of MazF-sth and 5 pmol of MazE-ec; lane 6, 1 pmol of MazF-sth and 10 pmol of MazE-ec; lane 7, 10 pmol of MazE-ec.

Though MazF-sth exhibited similarities in amino acid and cleavage sequences with MazF-bs, relatively fewer identities and similarities were found among their antitoxin MazEs. Accordingly, the homology is further discussed here by examining the neutralization of MazF-sth by non-cognate MazE-bs and MazE-ec. A decrease in activity was not observed when MazE-ec was pre-incubated with MazF-sth, whereas MazF-sth enzymatic activity was inhibited by MazE-bs in a concentration-dependent manner (Figure 5). The suppression was further examined by a fluorometric assay with the chimeric oligo probes UACAUA for MazF-sth and UACAU for MazF-bs (Figure S4). When MazFs were pre-mixed with cognate MazEs, the fluorescence intensity of the samples was greatly reduced compared to the samples containing only MazF-sth or MazF-bs. When MazF-sth or MazF-bs were pre-incubated with non-cognate MazE-bs or MazE-sth, respectively, the fluorescence increase in the samples was markedly slower than that for the enzyme-only samples (without MazE). Though such a fluorescence inhibition was not as strong as when either MazF-sth or MazF-bs was mixed with cognate-MazE, a cross-neutralization was readily observed, demonstrating that MazEF-sth and MazEF-bs share higher homology over MazEF-ec.

## 3. Discussion

This study characterized the MazF homolog conserved in *S. thermophilum* that specifically cleaves the UACAUA sequence, resembling the cleavage sequence of well-illustrated MazF-bs from *B. subtilis*, which can support the proliferation of MazF-sth’s host organism. Moreover, MazEF-sth also shared high similarities, in terms of amino acid sequences, with a collection of MazEF homologs, part of whose toxins had been revealed to cleave at the pentad motif UACAU. Proceeding from the cleavage sequences, the present study sought more commonalities in the physiological functions of these MazEF modules. Though no estimated target orthologous genes have been reported as targets for other MazFs, two predicted target genes of MazF-sth were found to be involved in the process of biofilm formation, which has also been specifically mentioned for several UACAU-cleaving MazF homologs [19,31,32]. As reported for MazF-bs, the pentad cleavage motif UACAU occurred significantly in two genes encoding surfactin synthetases, which play a significant role in biofilm formation [17]. Concerning MazEF conserved in *Clostridium difficile* (MazEF-cd), the susceptible target cell wall protein CwpV, which contains one UACAU motif, promotes biofilm-like aggregates or adhesion [19]. In Staphylococcus aureus, transcripts containing the cleavage sequence UACAU are not associated with biofilm formation. However, biofilm formation is enhanced when the gene encoding the MazF homolog is deleted [31,32]. It can therefore be hypothesized that these MazFs have homology and similarity in cleavage sequences and may play similar roles in biofilms [29,30]. Few of the mentioned analyses, however, were supported with tangible experiments. The markedly different distribution patterns of the above MazF cleavage sequences in the transcripts of their host bacteria should not be overlooked. The short cleavage sequence ACA of MazF-ec occurred multiple times among *E. coli* transcripts, leading to a global digestion pattern in cellular mRNAs [12]. Equally, the target sequence of MazF-bs, UACAU, also widely occurs within the *B. subtilis* genome, with a maximum of 17 repeats in a single gene [17]. Contrastingly, in the case of MazF-sth, it was observed that each of the 31 target CDSs contains only one UACAUA site. The determination of whether intracellular cleavage actually occurs remains to be covered.

While several models including stress responsory or persistence have been proposed for the physiological discussion of MazEF, few have been corroborated repeatedly [1,2]. In recent years, there has been a growing interest in turning to the evolution and genetic functions of the TA system [1,2,33,34,35,36], proposing that the module is likely to move between host organisms through horizontal transfer, as suggested by their heterogeneous distribution among genomics [2]. Since MazF-sth exhibited high similarities in amino acid sequences, as well as cleavage sequences, with multiple UACAU-like cleaving MazFs (e.g., MazF-bs), in this study, the relevance between MazEF-sth and MazEF-bs was further explored by examining MazF catalysis and MazEF neutralization. As two amino acids have been reported to be essential for MazF-bs activity, the MazF-sth mutants, Arg29 and Thr52, with site-directed mutagenesis at the corresponding conserved residues, lost most of the ribonuclease activity in an in vitro cleavage assay, indicating that there is a conserved catalysis mechanism between MazF-sth and MazF-bs. Moreover, in contrast to a growth assay that indicated no neutralization of MazF-bs by MazE-ec [17], it was found that the activity of MazF-sth was sequestered by non-cognate MazE-bs. The cross-neutralization implied that the MazEF modules, whose toxins cleave UACAU-like motifs, may share higher homology over other MazEF modules. In spite of the highly similar catalysis and neutralization mechanism between MazEF-sth and MazEF-bs, their host organisms displayed fewer phylogenic correlations. The phylogenetic analysis suggested that *Symbiobacterium* branched off during the earliest diversification of *Clostridia* species [37]. A revision to the phylum classification also positioned *Symbiobacterium* as a deep group within the class *Clostridia* as a result of its distinct genetic content compared to *Clostridia* [38,39]. However, through BLAST analysis by querying MazF-sth based on the TADB 2.0 in silico predicted data of the type II TA system (Dataset S1, S2), MazF-sth demonstrated a high degree of homology with many predicted toxins, including a considerable number of MazFs from *Clostridia* members and even MazFs from *Bacilli*-class bacterium [17,25]. For instance, MazF conserved in *Geobacillus thermodenitrificans* and MazF conserved in *C. difficile* scored the same among MazF-sth homologs, sharing 70% and 71% percent identity with MazF-sth, respectively, at query cover values of 95% and 100%. In contrast, *S. thermophilum*, a Gram-negative bacterium with 68.7% high G + C content showed much less genetic correlation with the Gram-positive *G. thermodenitrificans* classified as *Bacilli* or *C. difficile* with 29.04% G + C content [40]. Such a conflict between MazEFs amino acid sequence identities and their host organisms’ phylogeny implies the possibility of *mazEF-sth* acquisition by horizontal gene transfer.

Considering the above homology, the cross-neutralization between MazF and non-cognate MazE was examined, revealing the obvious suppression of MazE-bs on MazF-sth. The structure mechanism was analyzed by multiple sequence alignment (MSA) [11,13,14,16,17,18,19,20,25,26,41,42,43,44,45,46,47] (Figure S5) combined with the simulation of the MazEF-sth complex structure via AlphaFold2 [48] (Figure S6). Focus was placed on the interface; next, regions of the MazF-sth dimer interacting with MazE-sth were estimated as follows and highlighted with red/blue squares on MSA (Figures S5 and S6). (1) The loop connecting the β1 and β2 strands of MazF-sth (Gly22 to Arg29) interacted with Leu56 to Pro63 located at the C-terminal end of MazE-sth (Figure S6c). (2) The residues on the α1 helix and the following loop of MazF-sth (Asn36 to Pro44) interacted with Arg41 to Asn55 on MazE-sth (Figure S6d). (3) The loop between the β3 and β4 sheets of MazF-sth (residues from Thr52 to His63) interacted with Met40 to Glu61 at the α2 helix of MazE-sth (Figure S6e). (4) The loop between the β5 and β6 strands of MazF-sth (residues from Leu80 to Gln83) interacted with the side chain atoms of Tyr48, Met51, and Asn55 of MazE-sth (Figure S6f). (5) Pro61, Ile113, and Phe120 on one chain of MazF-sth and Val38 on the other chain of MazF-sth formed a hydrophobic groove, interacting with the inwardly directed hydrophobic residues Met40 and Leu44, presented on the α2 helix of MazE-sth (Figure S6g). Via MSA, these five regions that were presumed to be on the interface were found to be particularly conserved in the MazF homologs that cleave U^ACAU, including the MazF-bs, MazF-cd, MazF-sa, and the MazF in *Staphylococcus equorum*. In addition, regions (1), (2), and (5) were found to be conserved in MazFs that cleave U^ACAU-like sequences, such as MazF-da, which cleaves U^ACAAA, and MazF-hw, which cleaves UU^ACUCA. However, these regions are not conserved even locally in the amino acid sequences of MazF proteins known to cleave significantly different RNA sequences from UACAU (Figure S5). Accordingly, the restricted conserved residues corroborated the homology between some MazEFs and may be significant in determining the MazF cleavage sequences, providing more clues for the interaction between proteins and RNAs.

## 4. Conclusions

This study characterized the MazEF module conserved in syntrophic *S. thermophilum*, elucidating the specific cleavage of MazF-sth against the UACAUA ribonucleotide motif, which was notably similar to that of MazF-bs from the supporting *B. subtilis*. The intracellular targets of MazEF-sth were estimated, and the cross-neutralization between MazF-sth with non-cognate MazE-bs was discussed, elucidating the possibility of the *mazEF-sth* locus being an acquired DNA region.

The conserved nature of MazFs from some prokaryotes is highly relevant to their physiological functions in their host organisms and offers some clues with respect to genetic evolution. Our findings provide a foundation for further studies on the genetic development of MazEF toxin–antitoxins.

## 5. Materials and Methods

### 5.1. Plasmids, RNAs, and Oligonucleotides

The *mazE-sth* and *mazF-sth* gene sequences were obtained from TADB 2.0 in silico predicted toxin and antitoxin data in July 2021 (Dataset S1, S2) [25]. The coding sequences were inserted into pET-24a(+) vectors that carry T7 promotor sequences, plus an optional C-terminal His-Tag sequence. The recombinant plasmids were synthesized by GenScript Japan (Tokyo, Japan), and the codon of the CDS was optimized for enhanced expression in *E. coli*.

Synthetic RNA substrates were prepared as previously described [26,27]. The RNA barcode used for massive parallel sequencing was purchased from Japan Bio Services (Saitama, Japan). Fluorescently decorated oligonucleotide probes were purchased from Japan Bio Services (Table S2). All primers used in the PCR were purchased from Tsukuba Oligo Service (Ibaraki, Japan).

### 5.2. Protein Expression and Purification

The pET24a-*mazF-sth* and pET24a-*mazE-sth* plasmids were introduced into *E. coli* BL21(DE3) host cells (Biodynamics Laboratory, Tokyo, Japan). The transformed cells were cultured overnight on an LB plate containing 50 µg·mL^−1^ kanamycin at 37 °C. After 15 h of overnight pre-culture in a 20 mL selective LB medium containing 20 µg·mL^−1^ kanamycin, 20 mL cell suspension for MazF-sth or 10 mL suspension for MazE-sth was inoculated into 1 L kanamycin supplemented LB medium. When OD_600_ reached 3.0 for MazF-sth and 0.6 for MazE-sth, isopropyl β-D-1-thiogalactopyranoside, at a final concentration of 1 mM, was added into the LB medium to induce protein expression. After 3.5 h of induction, the cells were collected by centrifugation at 9200× *g* and stocked at −80 °C. For purification, the cell pellets were re-suspended with 20 mL of binding buffer (20 mM sodium phosphate buffer [pH 8.0], 500 mM NaCl, 0.05% Triton X-100, 5 mM β-mercaptoethanol, and 40 mM imidazole). The cell suspension was lysed using a Handy Sonic UR-20P (Tomy Seiko, Tokyo, Japan) and centrifuged. The supernatant was filtrated through a 0.45 µm membrane (Millex, Darmstadt, Germany). Then, the filtrate was loaded onto a 1 mL His-Trap FF column (Cytiva, Marlborough, MA, USA) for purification by AKTA pure 25 (Cytiva). The product was eluted with a high concentration of imidazole in elution buffer (20 mM sodium phosphate buffer [pH 8.0], 500 mM NaCl, 0.05% Triton X-100, 5 mM β-mercaptoethanol, and 500 mM imidazole), analyzed by sodium dodecyl sulfate polyacrylamide gel electrophoresis (SDS-PAGE) using e-PAGEL (Atto, Tokyo, Japan), and stained using CBB Stain One (Nacalai Tesque, Kyoto, Japan). Sample protein concentrations were derived using the Bio-Rad Protein Assay Kit (Bio-Rad, Hercules, CA, USA).

### 5.3. Cleavage Assay

To investigate the ribonuclease activity of MazF-sth, 0.5 pmol or 5 pmol MazF-sth was incubated at 25 °C for 10 min. For suppression by MazE-sth, 5 pmol of MazF-sth with 10 pmol or 50 pmol MazE-sth was mixed and incubated at 25 °C for 10 min. In addition, 50 pmol MazE-sth was solely incubated as a control experiment. Afterwards, each sample was treated with 300 ng synthetic RNA 1500-1 at 37 °C for 60 min in 30 µL of reactants. The reactants were then purified using RNA Clean and Concentrator^TM−5^ (Zymo Research, Irvine, CA, USA), and the eluted RNA fragments were separated on a polyacrylamide gel containing 7 M urea. The gel was stained with SYBR Gold (Life Technologies, Carlsbad, CA, USA) and scanned using a Typhoon 9210 imager (GE Healthcare, Chicago, IL, USA).

To identify the optimal temperature of MazF-sth, 0.1 pmol of MazF-sth was solely pre-incubated at 4 °C, 37 °C, 50 °C, 60 °C, 75 °C, or 90 °C for 10 min. Next, the samples were treated with 300 ng synthetic RNA 1500-1 and then incubated at the same temperature for 15 min. The RNA fragments were purified and analyzed using a 2100 bioanalyzer instrument (Agilent Technologies, Santa Clara, CA, USA) using the Agilent RNA 6000 Nano Kit (Agilent Technologies, Santa Clara, CA, USA).

For the cross-neutralization of MazF-sth by non-cognate MazE-ec and MazE-bs, 1 pmol of MazF-sth was incubated with 2, 5, or 10 pmol of each MazE for 10 min. Then, 1 pmol MazF-sth, MazEF mixture, or 10 pmol MazE were treated with 300 ng synthetic RNA 2000-1 at 37 °C for 60 min. The RNA fragments were purified and separated on a polyacrylamide gel containing 7 M urea. The gel was stained with SYBR Gold (Life Technologies) and detected using a Typhoon 9210 imager (GE Healthcare).

### 5.4. Massive Parallel Sequencing

The cleavage motif of MazF-sth was decided using a method previously developed by the present authors [26]. A total of 0.625 pmol of synthetic RNA substrate mixture of RNA 1000-1, L1000-1, 1500-1, L1500-1, H1500-1, 2000-1, L2000-1, and H2000-1 was fragmentated by 100 ng MazF-sth at 60 °C for 90 min. The samples were purified using RNA Clean and Concentrator^TM−5^ (Zymo Research). Next, these RNA fragments were phosphorylated with T4 polynucleotide kinase (TaKaRa, Kusatsu, Japan) and purified for ligation with a 45-nt barcode via T4 RNA ligase (TaKaRa). Afterwards, the barcoded RNAs were purified to exclude fragments shorter than 200 nt, and the concentrations of products were determined using the Qubit RNA Assay Kit (Life Technologies). Then, the cDNA library was constructed according to the NEB Ultra RNA Library Prep Kit for Illumina protocols (New England Biolabs, Ipswich, MA, USA) and finally loaded on the MiSeq platform (Illumina, San Diego, CA, USA) for sequencing.

The sequencing reads were analyzed using CLC Genomics Workbench version 12.0 (Qiagen, Hilden, German), while the barcode-ligated reads were extracted and mapped according to the known substrate sequences. To estimate the likelihood of each base acting as a clipping site, the RCI parameter, as an indicator of the cutting sites, was defined as the (n)^th^ coverage divided by that of the (n−1)^th^ position. When the coverage of the (n−1)^th^ position is 0, the RCI of the (n)^th^ position is defined equal to the coverage of the (n)^th^ position. The sites with the highest RCIs, as well as the 11 base motifs that centered on those sites, were collected and subsequently aligned and visualized using WebLogo.

### 5.5. FRET Assay

Twenty micromoles of fluorescently labeled probes (Table S2) were treated with 0.01 pmol MazF-sth. An equal amount of RNase A (Thermo Fisher, Vilnius, Baltics) was taken as a positive control. The samples were immediately loaded on a Light Cycler 480 (LC480) system (Roche, Basel, Switzerland) at 60 °C for 60 min, and the fluorescence intensity was recorded in real time. Parallel experiments were performed to collect triplicate data, and the average values were derived.

### 5.6. Site-Directed Mutagenesis and Cell-Free Expression

Site-directed mutagenesis was introduced into pET-24a(+)-*mazF-sth* plasmid containing MazF-sth CDSs. The codon encoding the 29th residue arginine (Arg29) and that for the 52nd residue threonine (Thr52) was replaced by the alanine codon using the PrimeSTAR^®^ Mutagenesis Basal Kit (TaKaRa). The linear DNA templates encoding R29A MazF-sth, T52A MazF-sth, and the wild type MazF-sth with a HisTag coding sequence at the 3′ end were amplified using KOD-PLUS-Ver.2 (Toyobo, Osaka, Japan). With the linear templates, the R29A MazF-sth, the T52A MazF-sth, and the wild type MazF-sth proteins were expressed using the cell-free expression system PUREfrex 2.0 (GeneFrontier, Kashiwa, Japan) and purified by using the Capturem^TM^ His-Tagged Purification Miniprep Kit (TaKaRa). The molecular weight and purity were examined with SDS-PAGE. The concentration of the products was measured via the Protein Assay Bradford method (Bio-Rad).

### 5.7. Accession Numbers

RNA 1000-1 (AB610944), RNA L1000-1 (LC659329), RNA 1500-1 (AB610949), RNA L1500-1 (LC659330), RNA H1500-1 (LC659334), RNA 2000-1(AB610950), RNA L2000-1(LC659331), and RNA H2000-1(LC659335).

## Figures and Tables

**Table 1:** Thirty-one genes containing one UACAUA motif.

Annotation	Locus Tag	Length (bp)	Product
Biofilm-related genes	RS16150	279	Veg family protein
	RS07500	669	PilZ domain-containing protein
Cell wall-related genes	RS17880	663	M23 family metallopeptidase
	RS05630	1098	polysaccharide pyruvyl transferase CsaB
	RS02655	1101	D-alanine--D-alanineligase
	RS16455	1275	UDP-N-acetylglucosamine1-carboxyvinyl transferase
	RS13520	1506	UDP-N-acetylmuramoyl-L-alanyl-D-glutamate—2,6-diaminopimelate ligase
Membrane proteins	RS08320	768	ABC transporter permease
	RS09880	1032	ABC transporter substrate-binding protein
	RS07025	1095	YeeE/YedE family protein
	RS12640	1797	ATP-dependent zinc metalloprotease FtsH
Metabolism	RS06785	636	guanylate kinase
	RS00715	813	NAD(P)binding domain-containing protein
	RS14505	1461	IMP dehydrogenase
Transcription and translation	RS16105	570	aminoacyl- tRNA hydrolase
	RS17470	624	helix-turn-helix transcriptional regulator
	RS09835	702	RNA polymerases sporulation sigma factor SigK
	RS16160	858	16SrRNA(adenine(1518)-N(6)/adenine(1519)-N(6))-dimethyl transferase RsmA
	RS05755	1227	tyrosine-tRNA ligase
	RS12140	2562	single-stranded-DNA-specific exonuclease RecJ
Stress response	RS07085	507	Hsp20/alpha crysalin family protein
DNA recombinant	RS09165	885	site-specific tyrosine recombinase XerD
Polysaccharides degradation	RS02780	1293	glycoside hydrolase family 18 protein
Unknown function	RS18735	210	hypothetical protein
	RS11335	246	hypothetical protein
	RS09245	447	hypothetical protein
	RS18275	558	hypothetical protein
	RS14425	666	hypothetical protein
	RS18470	672	hypothetical protein
	RS06565	921	hypothetical protein
	RS01700	921	hypothetical protein

## Data Availability

The sequencing dataset was deposited into the DDBJ Sequence Read Archive under the accession numberDRA015626.

## Supplementary Materials

The following supporting information can be downloaded at: https://www.mdpi.com/article/10.3390/toxins16020081/s1. Dataset S1: In silico predicted type II toxin from TADB 2.0 as of July 2021; Dataset S2: In silico predicted type II antitoxin from TADB 2.0 as of July 2021; Figure S1: Ribonuclease activity of MazF-sth at various temperatures; Table S1: Top 10 sites with the highest RCIs and their peripheral sequences; Table S2: Oligonucleotide probe sequences used in the fluorometric assay; Figure S2: Identification of MazF-sth specific cleavage sequence with 1 pmol of the enzyme; Figure S3: Purification and elution profiles of the wild type MazF-sth and MazF-sth mutants (R29A and T52A); Figure S4: Fluorometric assay of cross-neutralization; Figure S5: Amino acid sequences of MazF-sth and a collection of MazFs were aligned; Figure S6: Simulation of the MazEF-sth complex carried out by using AlphaFold2.
