# Supplementary material for: MazEF Homologs in Symbiobacterium thermophilum Exhibit Cross-Neutralization with Non-Cognate MazEFs
Source: Toxins (Basel). 2024 Feb 3;16(2):81. doi: 10.3390/toxins16020081 (PMC10893535; doi:10.3390/toxins16020081)
Supplement: Supplementary file 1 [file toxins-16-00081-s001.zip › toxins-2821569-SM.pdf]

Supplementary Materials

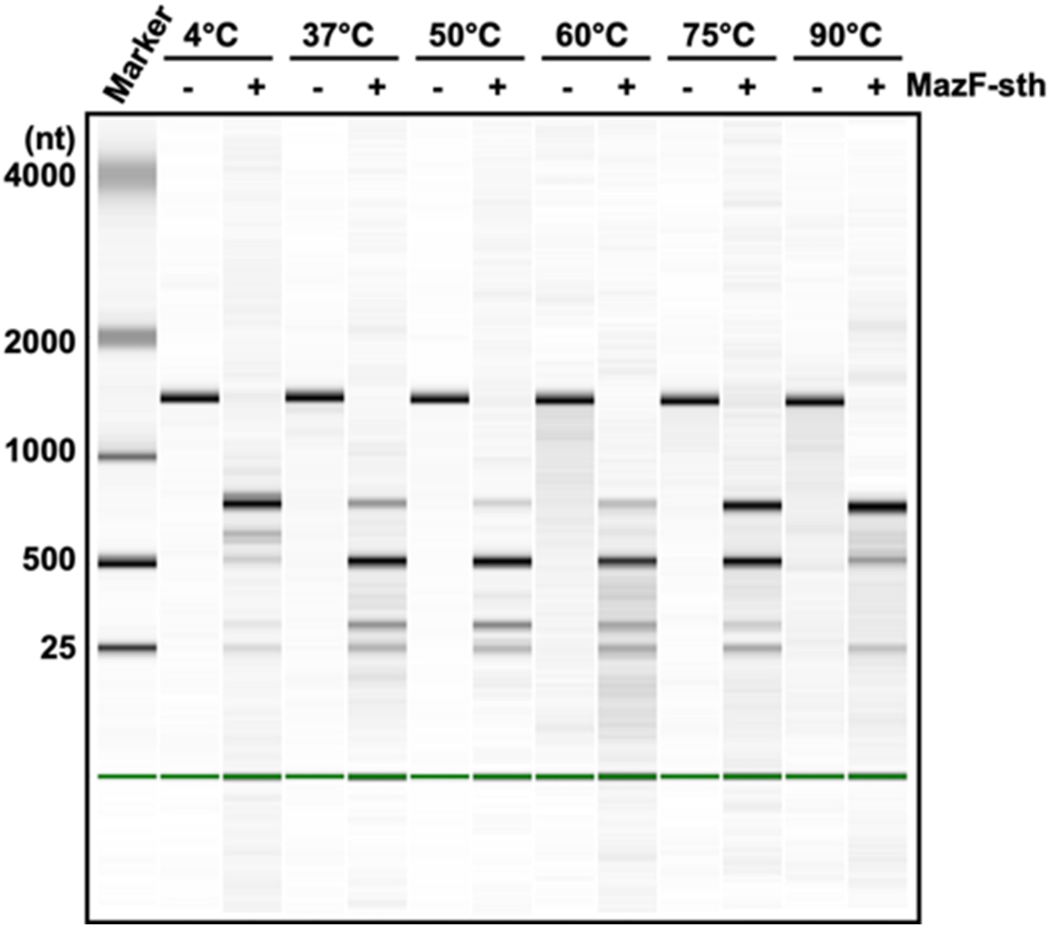

Figure S1. Ribonuclease activity of MazF-sth at various temperatures. MazF-sth solely was preincubated at 4 °C, 37 °C, 50 °C, 60 °C, 75 °C or 90 °C for 10 min. Samples were treated with 300 ng synthetic RNA 1500-1 (1533 nt) and incubated at the same temperature for 15 min.

Table S1. Top 10 sites with the highest RCI and their peripheral sequences

Rank	RNA	Position	RCI[1]	Coverage	Sequence[2]
1	H2000-1	1162	5406	5406	GCGGU^ACAUCG
2	L2000-1	1050	4026	4026	CCCUU^ACUUAA
3	H1500-1	543	3647	3647	CUGGU^ACAUAC
4	H2000-1	1451	2340	4680	ACGGU^ACAUAC
5	1500-1	767	1219	3657	UGAAU^ACAUAG
6	1500-1	961	884	1768	GACUU^ACCUAG
7	L1000-1	592	351	701	CUGUU^ACAUAU
8	2000-1	1572	328	328	ACCGU^ACAUCG
9	L2000-1	1803	1434	4463	UCAGU^ACGUAU
10	L1500-1	1098	142	6106	GUUGC^ACAUAA

[1] RCI is defined as the coverage of the $(n)^{th}$ position divided by the coverage of the $(n-1)^{th}$ position. When the coverage of the $(n-1)^{th}$ position is 0, the RCI of the $(n)^{th}$ position is corrected to the coverage of the $(n)^{th}$ position.

[2] ^ indicates the cleavage site, and underlined letter indicates the base with the highest RCI.

Table S2. Oligonucleotide probe sequences used in the fluorometric assay.

Name	Sequence [5′-3′]
DR-UACAUA	AAAAAUACAUAAAAAA
DR-AACAUA	AAAAAAACAUAAAAAA
DR-GACAUA	AAAAAGACAUAAAAAA
DR-CACAUA	AAAAACACAUAAAAAA
DR-UACAUU	AAAAAUACAUUAAAAA
DR-UACAUC	AAAAAUACAUCAAAAA
DR-UACAUG	AAAAAUACAUGAAAAA
DR-UACUUA	AAAAAUACUUAAAAAA
DR-UACCUA	AAAAAUACCUAAAAAA
DR-UACGUA	AAAAAUACGUAAAAAA

Underlined letters indicate RNA nucleotides, while all other letters indicate DNA nucleotides.

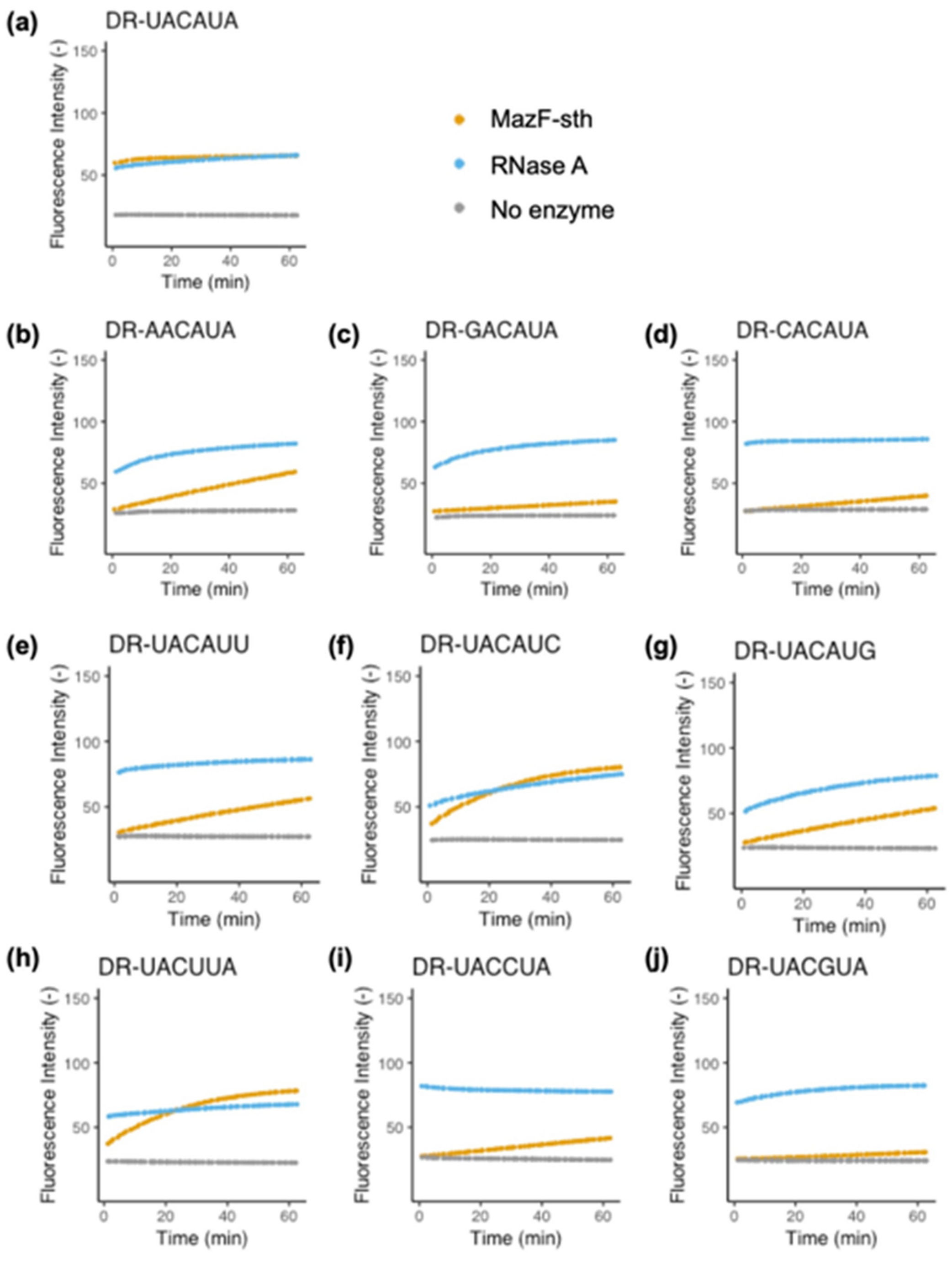

Figure S2. Identification of MazF-sth specific cleavage sequence with 1 pmol of the enzyme. MazF-sth (orange) was incubated with fluorometric probes at 60 °C for 60 min. Fluorescence intensities in the presence of RNase A (sky blue) and in the absence of enzymes (gray) were recorded as the control. Fluorescence intensities were measured in real time with each probe **(a)** DR-UACAUA, **(b)** DR-AACAUA, **(c)** DR-GACAUA, **(d)** DR-CACAUA, **(e)** DR-UACAUU, **(f)** DR-UACAUC, **(g)** DR-UACAUG, **(h)** DR-UACUUA, **(i)** DR-UACCUA, and **(j)** DR-UACGUA (Table S2).

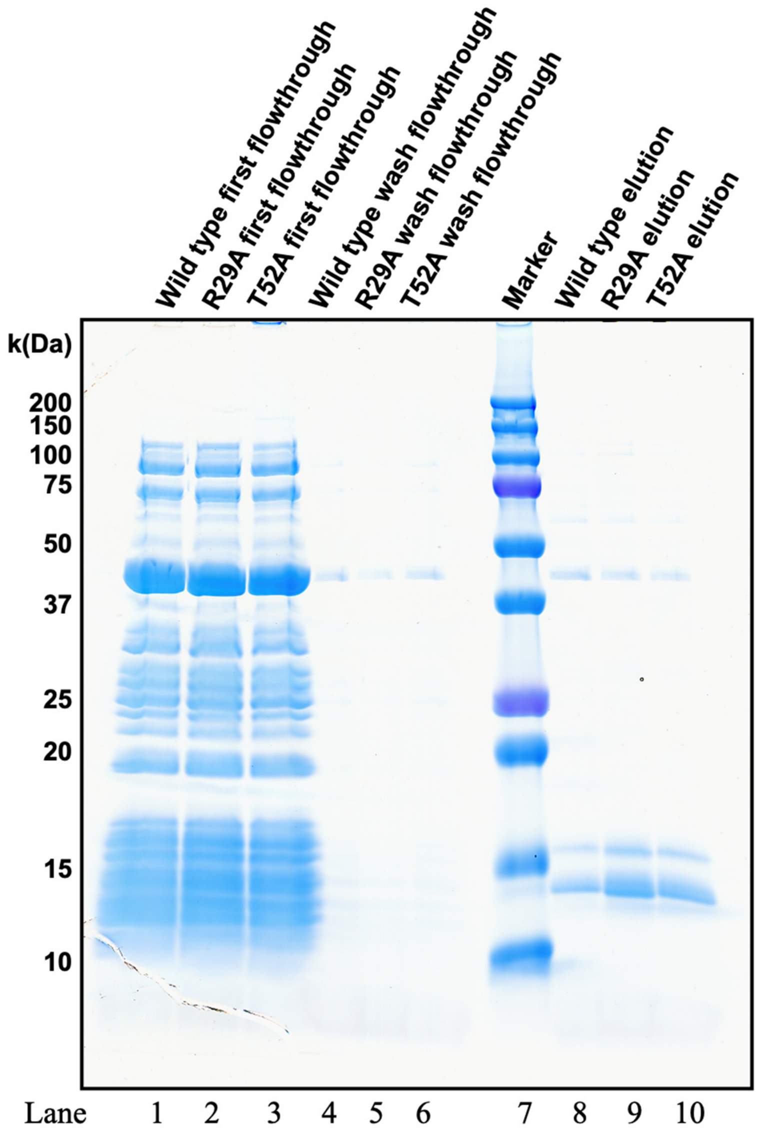

Figure S3. Purification and elution profiles of the wild-type MazF-sth and MazF-sth mutants (R29A and T52A). The cell-free expression production was initially loaded onto the affinity column tube. The first flowthrough of the sample was loaded onto the lanes 1 to 3. Lane 1, first flowthrough of wild-type MazF-sth; lane 2, first flowthrough of R29A MazF-sth; and lane 3, first flowthrough of T52A MazF-sth. The column membrane as well as the proteins bound thereto were washed with wash buffer (150 mM NaCl, 20 mM Na_3PO_4, pH 7.6). The flowthrough of the wash was loaded onto the lanes 4 to 6. Lane 4, wash flowthrough of wild-type MazF-sth; lane 5, wash flowthrough of R29A MazF-sth; and lane 6, wash flowthrough of T52A MazF-sth. The target proteins were eluted with the elution buffer (500 mM NaCl, 20 mM Na_3PO_4, 500 mM imidazole, pH 7.6). The elution was loaded onto the lane 8 to 10. Lane 8, elution of wild-type MazF-sth; lane 9, elution of R29A MazF-sth; and lane 10, elution of T52A MazF-sth. The bands were observed at the positions of theoretical molecular weight (approximately 14.9 kDa).

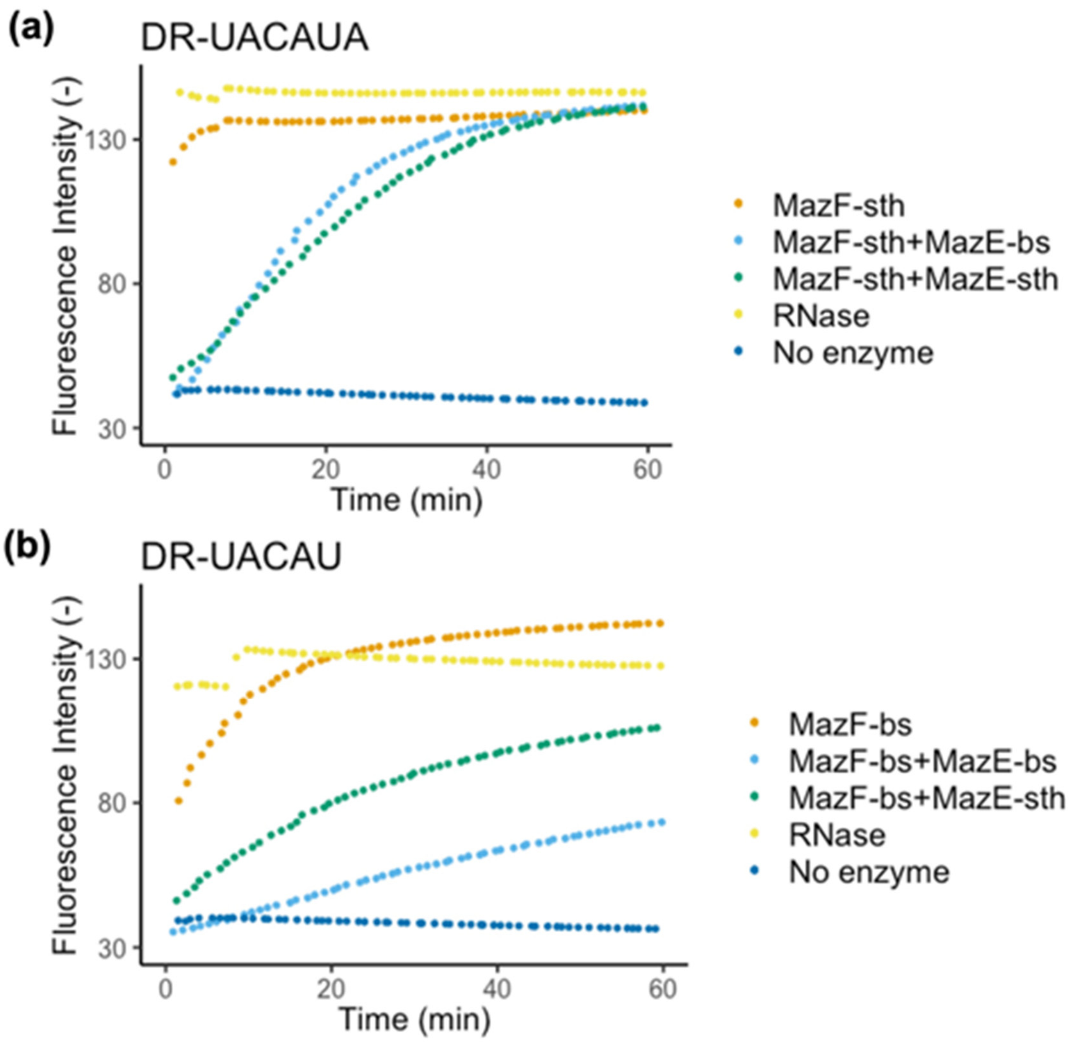

Figure S4. Fluorometric assay of cross-neutralization. (a) 0.1 pmol of MazF-sth (orange), and 0.1 pmol of MazF-sth pre-mixed with 10 pmol of MazE-bs (sky blue) or 10 pmol of MazE-sth (green) were incubated with the fluorometric probe (DR-UACAUA) at 37 °C for 60 min. The fluorescence intensity using the same probe in the presence of 50 pmol RNase A (Merck, Darmstadt, Germany) (yellow), as well as in the absence of enzymes (cyan cobalt blue) was recorded as a control. (b) 0.1 pmol of MazF-bs (orange), and 0.1 pmol of MazF-bs pre-mixed with 10 pmol of MazE-bs (sky blue) or 10 of pmol MazE-sth (green) were incubated with the fluorometric probe (DR-UACAU) at 37 °C for 60 min. The fluorescence intensity using the same probe in the presence of 50 pmol RNase A (yellow), as well as in the absence of enzymes (cyan cobalt blue) was recorded as a control.

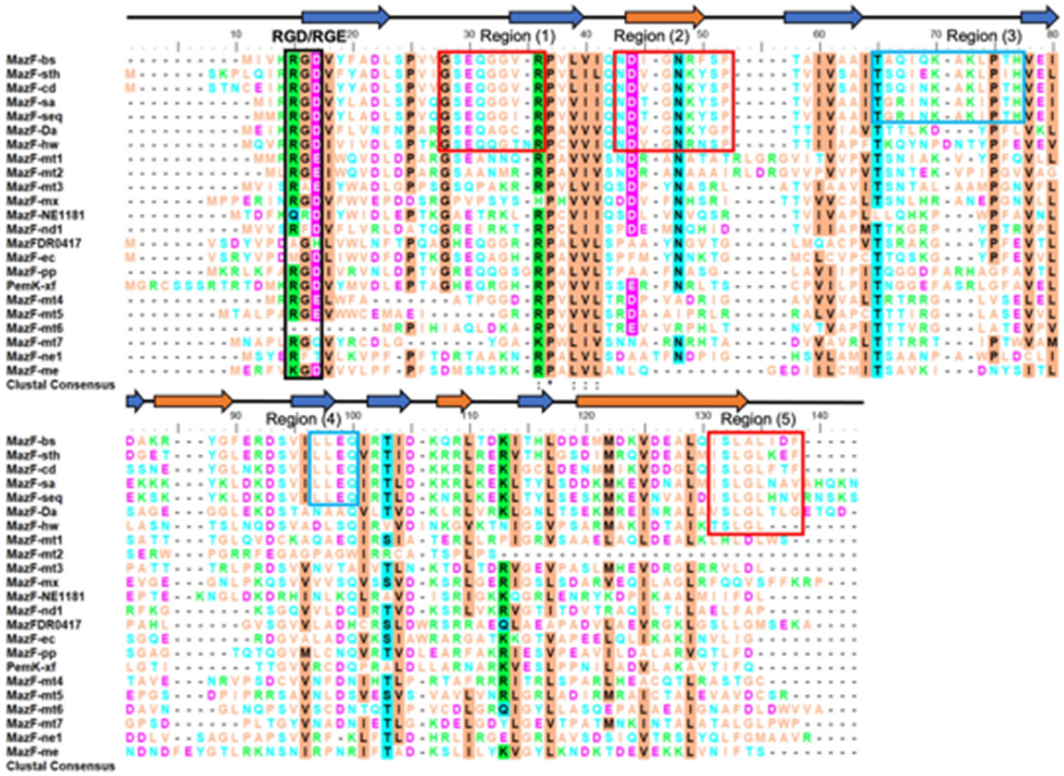

Figure S5. Amino-acid sequences of MazF-sth and a collection of MazF homologs are aligned, and specific cleavage sequences for the MazFs that have been uncovered are described in parentheses: bs, *Bacillus Subtilis* (UACAU) [1]; cd, *Clostridium Difficile* (UACAU) [2]; sa, *Staphylococcus Aureus* (UACAU) [3-5]; seq, *Staphylococcus Equorum* (UACAU) [4]; Da, *Candidatus* Desulforudis audaxviator (UACAAA) [6]; hw, *Haloquadra Walsby* (UUACUCA) [7]; mt1, mt2, mt3, mt4, mt5, mt6, and mt7, *Mycobacterium tuberculosis* (MazF-mt3, UUCCU and UUCCU; MazF-mt7, UCGCU) [8]; mx, *Myxococcus xanthus* (GUUGC) [9]; NE1181 and ne1, *Nitrosomonas europaea* (MazF-NE1181, AAU; MazF-ne1, UGG) [10, 11]; nd1, *Nitrospira* strain ND1 (AACU, AACG, and AAUU) [12]; DR0417, *Deinococcus radiodurans* (UACA) [13]; ec, *Escherichia coli* (ACA) [14]; pp, *Pseudomonas putida* (UAC) [15]; xf, *Xylella fastidiosa* (UACU and UACG) [16]; and me, *Methanohalobium evestigatum* (CUGGU and UUGGU) [17]. An asterisk (*) indicates a position with a fully conserved residue. A colon (:) indicates a highly conserved position between groups of strongly similar characteristics. Arrows indicate the predicted secondary structure; blue is beta-sheet, and orange is alpha-helix. The conserved residues are highlighted with a colored background and the colors of the background are in dependence on amino-acid chemical properties (green: positively charged residues, K, R, and H; magenta: negatively charged residues, D and E; salmon pink, hydrophobic residues, G, A, V, C, P, L, I, M, W, and F; cyan: polar residues, S, T, Y, N, and Q). The letters of the less conserved residues are in colored in the same manner instead of a colored background. The black square indicates the highly conserved RGD/RGE motif in almost all MazFs; blue and red squares highlight amino acids in MazF-sth that were assumed to interact with MazE-sth, corresponding to regions (1), (2), (3), (4), and (5), as described in Discussion. Blue squares, regions (3) and (4), marked amino acids conserved in UACAU-cleaving MazFs (MazF-bs, MazF-cd, MazF-sa, and MazF-seq); and red squares mark regions (1), (2), and (5), amino-acid sequences that are conserved in UACAU-like-cleaving MazFs (MazF-da [UACAAA], and MazF-hw [UUACUCA]).

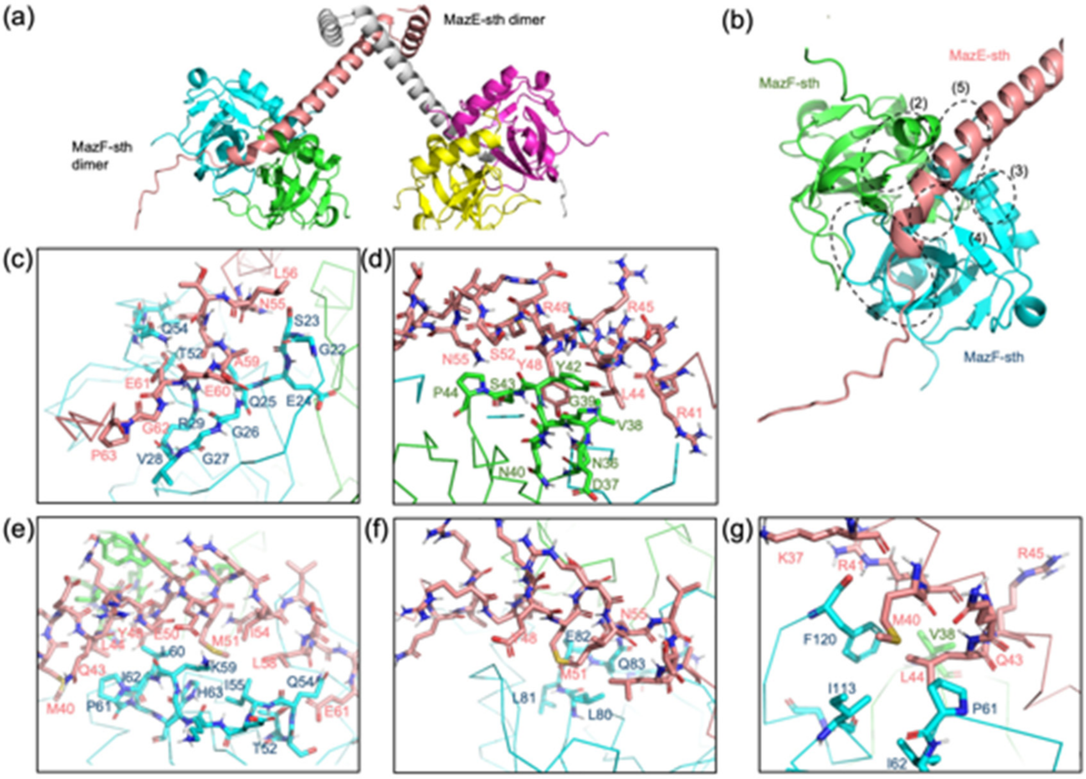

Figure S6. Simulation of the MazEF-sth complex carried out by using AlphaFold2 in: **(a)** overall structure of (MazF)2-(MazE)2-(MazF)2 complex; **(b)** interaction region between MazF-sth dimer (light green and cyan) and MazE-sth (salmon pink), on which the estimated interface (1)–(5) as described in Discussion are highlighted with dashed circles, and the amino-acid residues around the corresponding interaction interface in panels **(c)**–**(g)**, respectively.

References

1. Park, J. H.; Yamaguchi, Y.; Inouye, M. *Bacillus subtilis* MazF-bs (EndoA) is a UACAU-specific mRNA interferase. *FEBS Lett.* **2011**, *585*, 2526–2532.
2. Rothenbacher, F. P.; Suzuki, M.; Hurley, J. M.; Montville, T. J.; Kirn, T. J.; Ouyang, M.; Woychik, N. A. *Clostridium difficile* MazF toxin exhibits selective, not global, mRNA cleavage. *J. Bacteriol.* **2012**, *194*, 3464–3474.
3. Zhu, L.; Inoue, K.; Yoshizumi, S.; Kobayashi, H.; Zhang, Y.; Ouyang, M.; Kato, F.; Sugai, M.; Inouye, M. *Staphylococcus aureus* MazF specifically cleaves a pentad sequence, UACAU, which is unusually abundant in the mRNA for pathogenic adhesive factor SraP. *J. Bacteriol.* **2009**, *191*, 3248–3255.
4. Schuster, C. F.; Park, J. H.; Prax, M.; Herbig, A.; Nieselt, K.; Rosenstein, R.; Inouye, M.; Bertram, R. Characterization of a *mazEF* toxin-antitoxin homologue from *Staphylococcus equorum. J. Bacteriol.* **2013**, *195*, 115–125.
5. Xie, Y.; Wei, Y.; Shen, Y.; Li, X.; Zhou, H.; Tai, C.; Deng, Z.; Ou, H. Y. TADB 2.0: an updated database of bacterial type II toxin-antitoxin loci. *Nucleic Acids Res.* **2018**, *46*, D749–D753.
6. Tamiya-Ishitsuka, H.; Tsuruga, M.; Noda, N.; Yokota, A. Conserved Amino Acid Moieties of *Candidatus* Desulforudis audaxviator MazF Determine Ribonuclease Activity and Specificity. *Front. Microbiol.* **2021**, *12*, 748619.
7. Yamaguchi, Y.; Nariya, H.; Park, J. H.; Inouye, M. Inhibition of specific gene expressions by protein-mediated mRNA interference. *Nat. Commun.* **2012**, *3*, 607.
8. Zhu, L.; Sharp, J. D.; Kobayashi, H.; Woychik, N. A.; Inouye, M. Noncognate *Mycobacterium tuberculosis* toxin-antitoxins can physically and functionally interact. *J. Biol. Chem.* **2010**, *285*, 39732–39738.
9. Nariya, H.; Inouye, M. MazF, an mRNA interferase, mediates programmed cell death during multicellular *Myxococcus* development. *Cell* **2008**, *132*, 55–66.
10. Miyamoto, T.; Yokota, A.; Ota, Y.; Tsuruga, M.; Aoi, R.; Tsuneda, S.; Noda, N. *Nitrosomonas europaea* MazF specifically recognises the UGG motif and promotes selective RNA degradation. *Front. Microbiol.* **2018**, *9*, 2386.
11. Miyamoto, T.; Yokota, A.; Tsuneda, S.; Noda, N. AAU-specific RNA cleavage mediated by MazF toxin endoribonuclease conserved in *Nitrosomonas europaea. Toxins* **2016**, *8*, 174.
12. Aoi, R.; Miyamoto, T.; Yokota, A.; Ota, Y.; Fujitani, H.; Tsuneda, S.; Noda, N. MazF endoribonucleolytic toxin conserved in *Nitrospira* specifically cleaves the AACU, AACG, and AAUU motifs. *Toxins* **2020**, *12*, 287.
13. Miyamoto, T.; Ota, Y.; Yokota, A.; Suyama, T.; Tsuneda, S.; Noda, N. Characterization of a *Deinococcus radiodurans* MazF: A UACA-specific RNA endoribonuclease. *Microbiologyopen* **2017**, *6(5)*, e00501.
14. Zhang, Y.; Zhang, J.; Hoeflich, K. P.; Ikura, M.; Qing, G.; Inouye, M. MazF cleaves cellular mRNAs specifically at ACA to block protein synthesis in *Escherichia coli. Mol. Cell* **2003**, *12*, 913–923.
15. Miyamoto, T.; Kato, Y.; Sekiguchi, Y.; Tsuneda, S.; Noda, N. Characterization of MazF-mediated sequence-specific RNA cleavage in *Pseudomonas putida* using massive parallel sequencing. *PLOS ONE* **2016**, *11*, e0149494.
16. Lee, M. W.; Rogers, E. E.; Stenger, D. C. *Xylella fastidiosa* plasmid-encoded PemK toxin is an endoribonuclease. *Phytopathology* **2012**, *102*, 32–40.

17. Ishida, Y.; Inouye, K.; Ming, O.; Inouye, M. A CUGGU/UUGGU-specific MazF homologue from *Methanohalobium evestigatum*. *Biochem. Biophys. Res. Commun.* **2019**, *518*, 533–540.
